# Motor Neuron Diseases and Neuroprotective Peptides: A Closer Look to Neurons

**DOI:** 10.3389/fnagi.2021.723871

**Published:** 2021-09-17

**Authors:** Emanuela Zuccaro, Diana Piol, Manuela Basso, Maria Pennuto

**Affiliations:** ^1^Department of Biomedical Sciences, University of Padua, Padua, Italy; ^2^Veneto Institute of Molecular Medicine, Padua, Italy; ^3^Padova Neuroscience Center, Padua, Italy; ^4^Department of Cellular, Computational and Integrative Biology – CIBIO, University of Trento, Trento, Italy

**Keywords:** motor neuron (MN), IGF-1 (insulin-like growth factor 1), PACAP (pituitary adenylate cyclase-activating polypeptide), motor neuron disease (MND), ALS (Amyotrophic lateral sclerosis), spinobulbar muscular atrophy (SBMA), spinal muscular atrophy (SMA)

## Abstract

Motor neurons (MNs) are specialized neurons responsible for muscle contraction that specifically degenerate in motor neuron diseases (MNDs), such as amyotrophic lateral sclerosis (ALS), spinal and bulbar muscular atrophy (SBMA), and spinal muscular atrophy (SMA). Distinct classes of MNs degenerate at different rates in disease, with a particular class named fast-fatigable MNs (FF-MNs) degenerating first. The etiology behind the selective vulnerability of FF-MNs is still largely under investigation. Among the different strategies to target MNs, the administration of protective neuropeptides is one of the potential therapeutic interventions. Pituitary adenylate cyclase-activating polypeptide (PACAP) is a neuropeptide with beneficial effects in many neurodegenerative diseases, including Alzheimer’s disease, Parkinson’s disease, and more recently SBMA. Another neuropeptide that has a neurotrophic effect on MNs is insulin-like growth factor 1 (IGF-1), also known as somatomedin C. These two peptides are implicated in the activation of neuroprotective pathways exploitable in the amelioration of pathological outcomes related to MNDs.

## Introduction

The central nervous system (CNS) is populated by an incredible diversity of neuronal subtypes. Distinct neuron types are located in different parts of the organism and exhibit unique functions, depending on the very specific and finely controlled connectome (Molyneaux et al., 2009; Lodato and Arlotta, 2015). Furthermore, neurons of different types co-exist within the same region of the CNS and interplay to explicate high order functions, such as running, thinking or playing a piano (Finlay and Darlington, 1995). In the cerebral cortex, an astonishing diversity of excitatory glutamatergic projection neurons and inhibitory GABAergic interneurons, which release the gamma-aminobutyric acid neurotransmitter, have their specialized anatomy, morphology and physiology, and are coordinately assembled in a local microcircuitry (Markram et al., 2004; Molyneaux et al., 2005, 2009; Lodato et al., 2015). Likewise, the spinal cord is populated by multiple neuronal subtypes that have been classified over the last few decades according to their target and physiological properties. By the interplay of different neuron types, the motor system exhibits a vast range of activities, spanning from routine actions, such as walking or grasping, to more sophisticated movements, such as dancing or playing a piano (Hollyday et al., 1977; Landmesser and Pilar, 1978; Stifani, 2014). The establishment of the correct connections within the motor circuitry is critical for balanced electrical activity and motor function (Arber, 2012). Consequently, dysgenesis or dysfunction of the MN circuitry is associated with neurodegenerative and neuromuscular diseases, such as ALS, SBMA, and SMA. Although every year millions of people are diagnosed with highly debilitating and fatal neurodegenerative diseases, only few symptomatic treatments are currently available (Kanning et al., 2010). How this fine diversity in neuronal subtype identities and specific connectivity are assigned and what are the molecular logics underlying this process are still largely unknown, hampering the discovery of molecular candidates for treatment. In this review we describe the neuronal types affected in the most common forms of MNDs and the potential therapeutic strategies available based on the use of peptides with neuroprotective and neurotrophic effects.

## The Motor System

**FIGURE 1:**
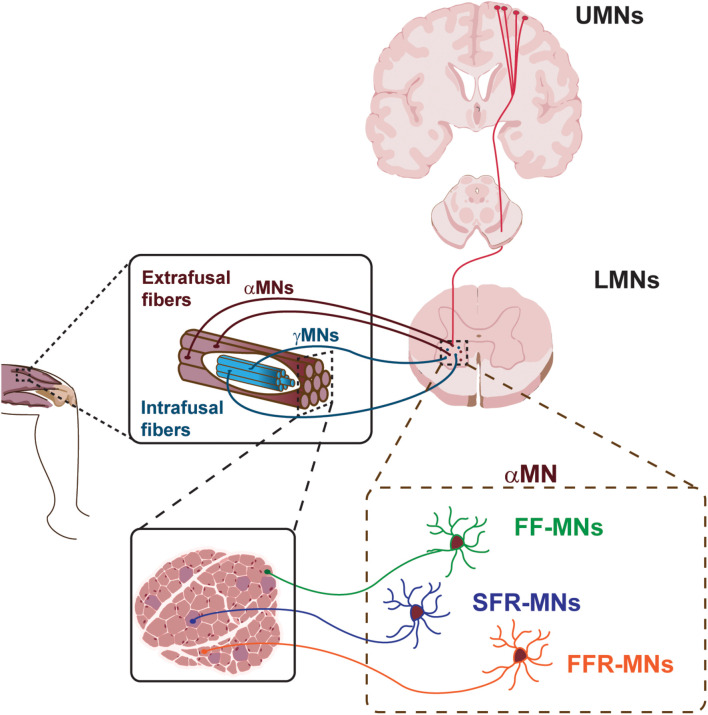
Schematic of the motor system. Upper motor neurons (UMNs) project to lower motor neurons (LMNs) in the brainstem and spinal cord. LMNs innervate skeletal muscle fibers. Different classes of α-MNs innervate diverse skeletal muscle fibers. FF-MNs, fast-fatigable MNs. SFR-MNs, slow fatigue resistant MNs. FFR-MNs, fast fatigue resistant MNs.

The voluntary movement is controlled by a highly complex system named motor system (Figure 1). It consists of the pyramidal and extrapyramidal systems and is composed of upper motor neurons (UMNs), lower motor neurons (LMNs), and interneurons. The motor impulses originate in the UMNs, which are specialized neuronal cells that reside in the cerebral cortex and propagate through their long axons to the brainstem and spinal cord. Here, they synapse on LMNs, whose axons project out of the CNS to directly or indirectly control skeletal muscles in the periphery. Each MN residing in the spinal cord can synapse with multiple muscle fibers at the periphery. The MN and all the skeletal muscle fibers it innervates through its axonal terminals constitute the motor unit.

## Upper Motor Neurons (UMNs)

UMNs are large pyramidal cells residing in the layer 5 of the cerebral cortex (Figure 1). They are responsible for the initiation of most voluntary movement by sending information down to the spinal cord through the corticospinal tract and directly activating interneurons or LMNs, which in turn signal on skeletal muscles for inhibiting or stimulating contraction, respectively. The direct pathway between the cerebral cortex and the brainstem/spinal cord is granted by the pyramidal tract, in contrast with extra-pyramidal tracts which provide indirect pathways for the coordination of movement. The UMNs of the pyramidal tract reside around the motor area of the cerebral cortex and, in general, innervate the musculature on the contralateral side of the body. Given the somatotopic organization of the motor area, UMNs controlling facial musculature are located on the most lateral area of the cerebral hemisphere, while control of the legs takes a more medial position, as represented in the cortical homunculus (Jang, 2014; Hooks et al., 2018). UMNs exert their effect via LMNs.

## Lower Motor Neurons (LMNs)

LMNs can be hierarchically subdivided into different subtypes, based on their location in the brainstem and spinal cord, their physiological properties and specific targets (Figure 1). LMN classes are organized into groups reflecting both their origin and their function, and are further arranged into motor pools, where groups of LMNs are responsible for innervating a single skeletal muscle. Moreover, MNs belonging to the same motor pool can be subdivided into three distinct subgroups according to the type of fiber they innervate, namely alpha MNs (αMN), beta MNs (βMN), and gamma MNs (γMN) (Kanning et al., 2010). αMNs innervate extrafusal muscle fibers and are the key of muscle contraction. They are characterized by a large soma, receive direct input from proprioceptive sensory afferents, and are the MN type that first undergoes dysfunction and degeneration in neurodegenerative diseases (NDDs). γMNs innervate intrafusal muscle fibers and modulate the sensitivity of the muscle spindle. They have a smaller soma and do not exhibit any motor function, rather they modulate skeletal muscle contraction. Finally, βMNs are characterized by a small soma and innervate both types of fibers. Being poorly abundant compared to the other two already mentioned subtypes, βMNs are a less well-defined population (Kanning et al., 2010).

All LMN classes differentiate from a unique pool of progenitors expressing the homeobox protein NK-6 homolog A (NKX6.1), paired box homeotic gene-6 (PAX6), and oligodendrocyte lineage transcription factor 2 (OLIG2) (Novitch et al., 2001; Vallstedt et al., 2001). Retinoic acid plays a central role in the diversification of MN subtypes and contributes to the spinal cord columnar organization (Lee et al., 2009). Nevertheless, very little is known about the molecular process of diversification of distinct MN subtypes. The first evidence of discrimination between αMN and γMN subtypes at embryonic stage came only few years ago, from a study pioneered by Ashrafi et al. (2012), where they demonstrated that the secreted signaling molecule Wingless-type MMTV integration site family member 7A (WNT7A) is selectively expressed in γMNs in the mouse spinal cord by embryonic day E17.5 and continues to molecularly distinguish these two subtypes in early postmitotic neurons. Additionally, few postmitotic class-specific markers have been identified: the estrogen related receptor gamma (ERR3, ESRRG), GDNF family receptor alpha 1 (GFRA1), and the ATPase, Na^+^/K^+^ transporting alpha1 (ATP1A1), have been shown to be selectively restricted to γMNs during the first postnatal weeks (Friese et al., 2009; Shneider et al., 2009; Edwards et al., 2013), while high expression of the RNA binding protein NeuN (fox-1 homolog 3 or RBFOX3) is specific for αMN, becoming one of the few available criteria of distinction of these two broad MN classes (Friese et al., 2009).

The human body has more than 600 muscles, which work in a finely controlled and coordinated manner, enabling an incredible variety of behaviors. In order to maintain this coordination, αMNs have to retain the characteristic of the type of fibers they innervate (Figure 1). In fact, depending on the type of innervated extrafusal fiber, αMNs are further subdivided into three additional subtypes. Slow αMNs innervate slow-twitch fatigue resistant fibers (SFR αMNs) and are characterized by an oxidative metabolism, whilst fast αMNs target fast-twitch fast-fatigable fibers (FF αMNs), which display a glycolytic metabolism, and fatigue-resistant fibers (FFR αMNs), which retain a level of oxidative capacity (Burke et al., 1973). Furthermore, SFR αMNs are small in size and fire regularly to control postural muscles, whereas low-excitable FF αMNs are characterized by a large soma and exert great force, yet for only a brief period of activity (Kanning et al., 2010; Duchateau and Enoka, 2011). Typically, one skeletal muscle contains all types of fibers innervated by their corresponding αMNs. However, the ratio between slow and fast fiber types varies between muscles, so as to suit each muscle’s function. As the MNs themselves, also the motor unit can vary in size. SFR αMNs innervate few fibers, constituting a small motor unit, that in turn generates small force for a sustained muscle contraction. Large FF αMNs can synapse with many fibers generating powerful and large motor units that ultimately enable massive contraction force, even if for a limited time. Finally, the motor unit composed of FFR αMNs display intermediate size and force generation. These differences in muscle fiber composition and contraction properties enable the execution of different functions and activities.

## Know the Target: Address Alpha Motor Neuron Diversity to Tackle MNDs

MNDs are characterized by the progressive dysfunction, degeneration and death of MNs. Although distinct MNDs have their own genetic and/or environmental cause, onset and prognosis, the vulnerability and consequent dysfunction and ultimately loss of MNs is a common denominator. MNDs can be classified into diseases primarily affecting either UMNs, LMNs, or both. ALS, also known as Lou Gehrig disease, is the most common late-onset MND characterized by the selective degeneration of both UMNs and LMNs. Most ALS cases are sporadic, and only 5–10% of ALS cases are considered to be familial. The disease manifests with progressive skeletal muscle weakness and atrophy, spasticity and fasciculation, finally leading to premature death. Primary lateral sclerosis (PLS) is a rare, idiopathic neuromuscular disease that often spontaneously occurs after the age of 50. It is characterized by the progressive weakness of voluntary movements as a result of the selective degeneration of UMNs, without impairment of LMNs. It is not a fatal disease, and individuals with PLS are treated only symptomatically. SMA involves the selective degeneration of LMNs, while no evidence of degeneration of UMNs has been reported. SMA is characterized by progressive muscle wasting that often results in early death. SBMA, also known as Kennedy’s Disease, is a neuromuscular diseases characterized by loss of LMNs and skeletal muscle atrophy. SBMA fully manifests in males, with a disease onset ranging from 18 to 64 years of age. The slow progression of the disease does not lead to premature death, but affected subjects are at risk of choking on food and aspiration pneumonia because of weakness of the bulbar muscles.

Several studies report that there is a gradient of vulnerability to MNDs among distinct αMN subtypes, with the low-excitable FF αMN class degenerating first (Nijssen et al., 2017). Why there is this kind of preferential susceptibility of FF αMN to MNDs and what are the molecular mechanisms driving this phenomenon are still largely unknown. Aimed to answer these questions, many efforts have been engaged by numerous groups, with the ultimate goal to shine the light on the key players and the molecular pathways involved in the divergence of αMN classes. These works resulted in the identification of genes whose expression is enriched in a specific subtype, such as synaptic vesicle 2a (SV2A), and ESSRB for postnatal SFR αMNs (Chakkalakal et al., 2010; Enjin et al., 2010), and calcitonin-related polypeptide alpha (CALCA), chondrolectin (CHODL), the non-canonical Notch ligand delta-like homolog 1 (DLK1), and mmp-9 for FF αMNs (Enjin et al., 2010; Kaplan et al., 2014; Muller et al., 2014). These markers, along with the soma size, firing properties and excitability, were the only criteria of distinction between αMN subtypes. Only recently, with the advent of single cell technology, MN diversity and subtype identity in the spinal cord is starting to be addressed, shading light on their unique transcriptomic profile (Delile et al., 2019; Alkaslasi et al., 2021; Blum et al., 2021). In particular, Blum et al. (2021) have profiled the transcriptome of 43,890 single-nuclei and identified distinct MN clusters in the autonomic and somatic motor system. In particular, they identified 16 subpopulations of sympathetic visceral MNs, including several clusters that localize to the sacral spinal cord, as well as a new skeletal MN population, which shares characteristics of the βMNs (Blum et al., 2021). Interestingly, Blum et al. (2021) deciphered the gene expression modules which define the differences between SFR, FFR, and FF MNs, using segmentation with known markers followed by hierarchical clustering. They identified novel transcriptional markers for FF and SFR populations, Kcnq5 and Prkcd, respectively, widening the molecular tools that can be exploited to study specifically α-MN subpopulations. Alkaslasi et al. (2021) have further performed single-nucleus RNA sequencing of cholinergic neurons, revealing distinct subpopulations characterized by specific molecular profiles. Taken together, these studies have further our knowledge on the unique molecular profiles of MN subtypes, which is instrumental for disease study and modeling.

## LMN Vulnerability to MNDs

Both in mouse and human, there is a selective vulnerability of different subtypes of MNs, with specific populations being particularly affected and dying very early, whilst others surviving, even at late stages of the disease. Spinal MNs innervating skeletal muscles undergo progressive and massive dysfunction at early stages of disease, causing the gradual loss of nearly all voluntary movements. On the other hand, those neurons innervating specific facial muscles, such as extra-ocular muscles, are the last neurons to be affected. Moreover, it has been shown that eye movement and sexual functions remain relatively unimpaired in ALS patients, even at the final stages of the disease (Kanning et al., 2010). Given that many disease-triggering mutant proteins described to date, such as the androgen receptor (AR), superoxide dismutase 1 (SOD1), and TAR-DNA binding protein 43 (TDP-43), are widely expressed in neurons and non-neuronal cells, the hypothesis that this selective vulnerability is due to their unique transcriptomic background has been largely investigated. Many groups have indeed focused their research on uncovering common patterns of selective protection or vulnerability in different MNDs, thereby identifying transcriptional changes between susceptible and resistant MN pools (Hedlund et al., 2010; Brockington et al., 2013; Kaplan et al., 2014). These efforts have led Kaplan et al. (2014) to elegantly show that the matrix metalloproteinase-9 (mmp-9) is strongly expressed in spinal MNs, while absent from oculomotor and Onuf’s nuclei, becoming a therapeutic candidate for ALS treatment. This work underscores the importance of decoding the transcriptional landscapes of vulnerable and resistant MN subtypes, opening new perspectives in the identification of novel therapeutic targets. Decoding the mechanistic principles that shape neuronal diversity in spinal MNs is among the fundamental steps required to uncover the molecular substrates of the selective vulnerability underlying the pathogenesis of neurodegenerative diseases and to elaborate successful regenerative therapies in the near future.

## Therapeutic Strategies for MNDs

To date, no effective cure is available to stop or reverse the progressive symptoms of MNDs. Patients receive supportive treatments to maintain their quality of life, such as physical rehabilitation to help muscles maintaining posture and slow atrophy. Moreover, few compounds have been successfully tested and approved for treatment of MNDs. Riluzole was for a long time the only approved drug from U. S. Food and Drug Administration (FDA) to treat ALS (Bensimon et al., 1994). This drug increases patient survival by 10% in clinical trials, probably protecting MNs by reducing the release of glutamate and blocking sodium channels. In 2017, the FDA approved Edavarone as therapeutic strategy to treat ALS. Edavarone is an antioxidant drug that slows disease progression measured through the evaluation of motor functions in patients (Writing Group; Edaravone (MCI-186) ALS 19 Study Group, 2017). For the treatment of SMA two drugs have been approved by FDA: Nusinersen and onasemnogene abeparvovec-xioi. Nusinersen is an anti-sense oligonucleotide (ASO)-based treatment that affects the splicing of *SMN2* gene, increasing full length SMN protein levels and ameliorating motor function (Passini et al., 2011; Hoy, 2017). Onasemnogene abeparvovec-xioi is a later approved adeno-associated virus vector-based gene therapy that acts introducing a new copy of *SMN1* gene in patients, stabilizing motor symptoms and improving walk capacity (Mendell et al., 2017). However, none of the mentioned therapeutic approaches is broadly applicable, thus justifying and encouraging ongoing research.

**FIGURE 2:**
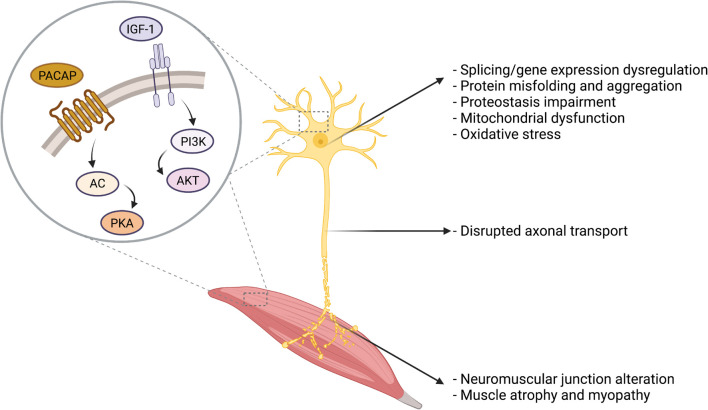
Overview of the cellular and molecular alterations in MNDs. Several cellular and molecular alterations are common to distinct MNDs, including protein misfolding and aggregates, proteostasis impairment, oxidative stress, and mitochondrial dysfunction. IGF-1 and PACAP main signal transduction pathways are presented in the figure.

Among the different therapeutic strategies under investigation to treat MNDs, the administration of neuroprotective peptides is an appealing approach that may address common molecular pathways dysregulated in the degenerating MNs (Table 1). Distinct MNDs have indeed distinct etiology, onset, manifestation, and progression. However, they also undergo common dysfunction that ultimately leads to neuronal degeneration, including protein misfolding and aggregation, proteostasis impairment, mitochondrial dysfunction, and gene expression dysregulation, as summarized in Figure 2. In the next paragraphs, two neuropeptides with promising therapeutic potential in MNDs will be discussed, namely IGF-1 and PACAP (Figure 2).

**TABLE 1:** Effects of PACAP and IGF-1 on motor neurons and their role in MNDs.

Effects on motor neurons	References
**IGF-1**	
Promotion of neuronal survival *in vitro* and *in vivo*	Ang et al., 1992; Hughes et al., 1993
Enhancing of axonal sprouting	Caroni and Grandes, 1990
Protection against excessive glutamate and ischemia	Nakao et al., 2001; Vincent et al., 2004
Induction of nerve regeneration	Kanje et al., 1989

**Effects in MNDs**	**References**

Improved motor functions after intrathecal administration in ALS patients	Nagano et al., 2005
Improved muscle force, motor coordination and survival in SOD1-G93A ALS mice	Saenger et al., 2012; Wang et al., 2018
Reduction of MN degeneration and increase of neuromuscular junction innervation in a SMA mouse model	Tsai et al., 2014
Increased muscle fibers hypertrophy and the number of functionally active axonal sprouts in a SMA mouse model	Krieger et al., 2014
Improving of motor performances, attenuated weight loss and increased survival in a SBMA mouse model	Rinaldi et al., 2012
Amelioration of ionic current defects in a SBMA cell model	Jimenez Garduno et al., 2017

**Effects on neurons**	**References**

**PACAP**	
Promotion of neuronal survival	Dejda et al., 2011; Manecka et al., 2013
Induced in damaged neurons	Pettersson et al., 2014
Promotion of axonal regeneration	Tsuchida et al., 2014
**Effects in MNDs**	
Neuroprotective action on preganglionic parasympathetic and sympathetic neurons in SOD1-G93A ALS mouse model	Ringer et al., 2013
Increase of cell survival in a ALS cell model	Maugeri et al., 2019
Attenuation of apoptotic signaling in ALS iPSC-derived MNs	Bonaventura et al., 2018
MN protection in SBMA cell and mouse model	Polanco et al., 2016
Amelioration of ionic current defects in a SBMA cell model	Jimenez Garduno et al., 2017; Martinez-Rojas et al., 2021

## IGF-1: An Old Friend With Neuroprotective Effects

IGF-1 is a small, single-chain polypeptide encoded by the *IGF-1* gene, localized on chromosome 12, and encoding a pre-propeptide subsequently processed to produce the active peptide. IGF-1 has a structure similar to that of insulin, with a similarity of about 50%, despite its diversified functions. In fact, IGF-1 is a member of the insulin-related peptide family, which also includes insulin and IGF-2. The human *IGF-1* gene has 6 exons, by which several transcript variants are produced, leading to the generation of the mature 70-amino acid IGF-1 peptide (Oberbauer, 2013). In the brain, the proteolytic cleavage also gives rise to two additional active proteins: a 67-amino acid isoform lacking three amino acids at the N-terminal of the protein, and the tripeptide cleavage product. IGF-1 binds to type I IGF receptor (IGF-IR), which belongs to the receptor tyrosine kinase (RTK) family, and shares about 60% sequence homology with the insulin receptor. The functional receptor is a dimer, which can also partner with insulin receptor. Two pathways are activated upon binding of IGF-1 to its receptor, the PI3K/Akt and the RAS/RAF/MAPK pathways, with mitogenic, antiapoptotic and transforming outcomes.

IGF-1 was first identified in 1957, when it was described as the “sulfation factor” induced in blood to act as mediator of growth hormone (GH) effects in the rat cartilage (Salmon and Daughaday, 1957). IGF-1 is mainly expressed at all stages of development in the liver and is regulated by pulsatile pituitary GH secretion. Nevertheless, IGF-1 and its receptor are found to be expressed in all tissues, with autocrine and paracrine effects. In the central nervous system, IGF-1 and IGF-IR mRNA transcripts are both present in the grey matter, and the highest expression is seen in areas of high plasticity. IGF-1 was detected in E14-15 rat neurons, with a peak at postnatal day 14 (Anlar et al., 1999). In the developing rat spinal cord, IGF-1 mRNA was detected in neurons, particularly of the cervicothoracic spinal cord (Rotwein et al., 1988). In the rat adult spinal cord, IGF-1 is detected in the ventral horn, sympathetic and dorsal root ganglia (Andersson et al., 1988). In a comparative study between normal and ALS patient adult spinal cords, IGF-1 quantitative receptor autoradiography revealed several IGF-1 binding sites. In normal human spinal cord high concentrations of IGF-1 binding sites were detected in the dorsal horn and central canal, intermediate levels in the ventral horn, intermediate zone, lamina X and tractus of Lissauer, while low levels were reported in dorsal, lateral and ventral white matter (Adem et al., 1994). Also, the IGF-1 immunoreactivity was detected in the same area of IGF-IR.

IGF-1 exerts neurotrophic properties in different regions of the CNS. Its early expression in fetal and adult CNS underlies its central role in neuronal development and trophic maintenance with both paracrine and autocrine mechanisms (D’Ercole et al., 1996). The importance of IGF-1 in CNS development was demonstrated with the generation of homozygous IGF-1 null mice, in which reduced brain size, altered myelination and loss of hippocampal granule and striatal parvalbumin-containing neurons were observed (Beck et al., 1995; Ye et al., 2002). Consistently with the phenotype of the IGF-1 knockout mice, the overexpression of IGF-1 leads to larger brain and increased myelination (Carson et al., 1993). Intuitively, GH production decreases with age, provoking a significant drop in IGF-1 levels in all tissues of the body, comprising the CNS, probably contributing with the decline in cognitive function (Muller et al., 2012; Toth et al., 2015). In MNs, IGF-1 promoted neuronal survival *in vitro* and *in vivo* in a facial axotomy model (Ang et al., 1992; Hughes et al., 1993). Moreover, IGF-1 enhanced MN axonal sprouting on skeletal muscle cells *in vitro* (Caroni and Grandes, 1990). IGF-1 has neuroprotective effects on MNs, protecting them against different insults, such as excessive glutamate and ischemia (Nakao et al., 2001; Vincent et al., 2004). In lesioned sciatic nerves, it was shown that IGF-1 induces regeneration (Kanje et al., 1989). IGF-1 mRNA levels increase distal to the site of crush and decrease after axonal regeneration, suggesting a role for IGF-1 in the regenerative processes (Glazner et al., 1994).

## Role of IGF-1 in MNDs

Given its neuroprotective properties, IGF-1 has been extensively investigated as potential therapeutic for neurodegenerative diseases (Bryan and Bowman, 2017; Yang et al., 2018). Several studies have explored the potential therapeutic effect of IGF-1 in MNDs. In ALS, recombinant human IGF-1 (rh-IGF-1) entered two randomized double-blind placebo-controlled clinical trials (Lai et al., 1997; Borasio et al., 1998). In these clinical trials, rh-IGF-1 was administered subcutaneously for 9 months with no appreciable effects on ALS patients. Posterior meta-analysis studies revealed that rh-IGF-1 administration was beneficial with low evidence, without an improvement in survival (Beauverd et al., 2012). In another clinical trial subcutaneously injected hr-IGF-1 revealed no difference in primary or secondary outcomes, defined as manual muscle testing score and tracheostomy-free survival and ALS functional rating scale, respectively (Sorenson et al., 2008). The meta-analysis study by Beauverd et al. (2012) confirmed the lack of differences between placebo and rh-IGF-1-treated patients in all the outcomes in this clinical trial. In 2005, a double-blind clinical trial was performed to assess the effect of intrathecal administration of IGF-1 in 9 patients with ALS, revealing a positive effect in motor functions without any variations in vital capacity (Nagano et al., 2005). Taken together, the results of these clinical trials suggest the use of routes of administrations that circumvent the limitations of IGF-1 bioavailability and the need for further data derived from pre-clinical studies. In SOD1-G93A mice, a polyethylene glycol-modified IGF-1 was systemically administered, obtaining encouraging results on muscle force, motor coordination and survival of the milder phenotype line (Saenger et al., 2012). Wang et al. (2018) used the same ALS mouse model to conduct a pre-clinical study systemically administering the animals with adeno-associated virus (AAV) expressing IGF-1, finding a consistent improvement of survival.

IGF-1 therapeutic potential has also been tested in SMA. In a severe mouse model of SMA, the intravenous administration of AAV vectors encoding hr-IGF-1 at postnatal day 1 reduced MN degeneration and increased innervation at the neuromuscular junction after 7 days, and it prolonged the mouse survival (Tsai et al., 2014). Importantly, this approach increased survival of motor neuron (SMN) expression levels in spinal cord, brain and muscles. In a SMA mouse model with respiratory distress type 1, subcutaneous administration of IGF-1 increased muscle fibers hypertrophy and the number of functionally active axonal sprouts, without any effect on MN survival (Krieger et al., 2014). These studies suggest that IGF-1 is a promising adjuvant therapeutic route for SMA patients, but more experimental evidence needs to be provided.

The role of IGF-1 in SBMA pathogenesis was first investigated because of the ability of this molecule to activate the PI3K-AKT pathway (Palazzolo et al., 2007). AKT-dependent phosphorylation of serines 215 and 792 on polyglutamine-expanded androgen receptor impeded ligand binding, improving cell survival in MN-derived cells modeling SBMA. Thus, in this preliminary study, IGF-1 had beneficial effects on toxicity induced by the mutant protein. Muscle-specific IGF-1 overexpression in SBMA transgenic mice rescued behavioral and histopathological abnormalities, extending lifespan, through the induction of AKT-dependent polyglutamine-expanded androgen receptor protective phosphorylation (Palazzolo et al., 2009). To translate these findings into a possible treatment, a pre-clinical study was performed on transgenic SBMA mice, injecting intraperitoneally rh-IGF-1. The treatment improved motor performances, attenuated weight loss and increased survival (Rinaldi et al., 2012). The encouraging outcomes in this pre-clinical study provided the rationale for a double-blind, randomized, placebo-controlled, multicenter clinical trial in which 24 patients were injected intravenously with IGF-1 or placebo (Grunseich et al., 2018). This phase II clinical trials showed that IGF-1 administration preserved thigh muscle volume of SBMA patients, but did not affect muscle strength or function, suggesting the potential of IGF-1 with the need to deepen the study of its effects. Possibly, a combined treatment of IGF-1 together with an anti-androgen, such as leuprorelin (Banno et al., 2009), may enhance the potential of both approaches to either arrest or at least delay disease progression. Consistent with their neuroprotective effects, both IGF-1 and PACAP ameliorated ionic current defects in a cell model of SBMA (Jimenez Garduno et al., 2017; Martinez-Rojas et al., 2021). The promising therapeutic effects of these peptides may ultimately contribute to therapy development for MNDs.

## PACAP as a Promising Therapeutic Strategy for MNDs

Among the neuropeptide gene superfamily, PACAP belongs to the glucagon/secretin gene family, together with glucagon, secretin, vasoactive intestinal peptide, growth hormone releasing hormone, and gastric inhibitory peptide genes (Soles-Tarres et al., 2020). PACAP is synthetized by *ADCYAP1* gene as prepro-PACAP peptide precursor, which is then processed through proteolytic cleavage that gives origin to several active peptides, including PACAP-38 and PACAP-27 (Miyata et al., 1989). PACAP is the ligand of three heterotrimeric G protein-coupled receptors, namely ADCYAP Receptor Type 1 (PAC1), Vasoactive Intestinal Peptide Receptor 1 (VPAC1), and Vasoactive Intestinal Peptide Receptor 2 (VPAC2). The stimulation of these receptors leads mainly to activation of adenylyl cyclase (AC)/protein kinase A (PKA) signaling, but other cAMP-dependent pathways may play important roles in the downstream effects of PACAP (Zhou et al., 1999; Ster et al., 2007; Juhasz et al., 2015). PACAP was first identified in Miyata et al. (1989) as a regulatory peptide that increases AC activity in the anterior pituitary cells, observation from which they coined its name. Nevertheless, thirty years of studies revealed that PACAP is broadly expressed in the central nervous system as well as in peripheral tissues. In the rat spinal cord, PACAP mRNA is detected as early as E14 (Jaworski and Proctor, 2000), while in the adult mouse it is expressed in lamina 1 of the dorsal horn of the spinal cord, and in the lateral gray column (Condro et al., 2016). In addition, its expression was also detected in the human adult spinal cord in the dorsal and ventral horn, and especially in the thoracic segments in the lateral funiculus projecting into the intermediolateral cell column (Dun et al., 1996). These observations suggest that PACAP is involved in sensory and autonomic pathways. PAC1, VPAC1, and VPAC2 receptors are widely expressed in the central nervous system. The most studied receptor is PAC1, the only PACAP-specific receptor. PAC1 is expressed in the spinal cord starting from E14 through E18 (Jaworski and Proctor, 2000). Other studies revealed that both PAC1 and VPAC1 receptors are consistently expressed in MNs of the rat facial motor nuclei (Zhou et al., 1999), while VPAC2 has been recently shown to be expressed in αMNs (Blum et al., 2021). PACAP has been shown to bind with high affinity to PAC1 and VPAC2 receptors, promoting dendritic outgrowth of cortical neurons.

In the central nervous system, PACAP has a plethora of functions in development, aging and regeneration. It regulates neurite outgrowth (Ogata et al., 2015), neuropeptide and neurotransmitter production (Stroth et al., 2013) and, importantly, neuronal survival (Dejda et al., 2011; Manecka et al., 2013). Among the different PACAP functions, the ability to induce neuroprotection is well-studied in several fields, from neuroinflammation to neurodegeneration. In a model of peripheral nerve injury, PACAP is extensively induced in the damaged neurons and triggers the activation of neuroprotective pathways (Pettersson et al., 2014). Fang et al. (2010) analyzed the effect of PACAP administration in combination with mesenchymal stem cells in rats subjected to spinal cord injury, demonstrating a therapeutic potential for this neuropeptide. The beneficial effect of PACAP was also confirmed in another mouse model of spinal cord injury, highlighting that the neuropeptide may improve motor functions with the induction of axonal regeneration (Tsuchida et al., 2014).

## Role of PACAP in MNDs

PACAP effects were deeply investigated in NDDs, such as Parkinson’s disease and Alzheimer’s disease (Rat et al., 2011; Han et al., 2014; Maasz et al., 2017), as well as MNDs. In a widely used transgenic mouse model of ALS expressing SOD1 carrying the ALS-linked mutation, glycine 93 substitution to alanine (G93A), PACAP exhibits a neuroprotective action on vulnerable neurons. In particular, it has been reported that the genetic depletion of PACAP in SOD1-G93A mice led to a loss of preganglionic parasympathetic and sympathetic neurons—which normally constitutively express PACAP—while sparing somatomotor neurons, indicating that PACAP has a neuroprotective role on visceromotor neurons in ALS (Ringer et al., 2013). Moreover, in an *in vitro* model of ALS, PACAP treatment increased cell survival through the activation of PKA/EGFR/MMP-2 axis (Maugeri et al., 2019), while in motor cortex of ALS patients, PACAP and its receptor PAC1 mRNA levels were dysregulated (Bonaventura et al., 2018). In line with the emerging role of PACAP in MN survival, it has been reported that PACAP treatment attenuated apoptotic signaling activated in human induced pluripotent stem cells (iPSC)-derived MNs following starvation. Of note, PACAP has also been recently shown to attenuate SBMA phenotype in *in vitro* and *in vivo* models, implying that the neuroprotective effects of this neuropeptide extend to different types of neurological conditions involving the motor system. In the case of SBMA, PACAP activated a signaling pathway that ultimately resulted in the dephosphorylation of the disease-causing protein, polyglutamine-expanded AR, thus enhancing its degradation, leading to MN protection (Polanco et al., 2016). In a preclinical study, a novel potent and stable PACAP analog was administered to SBMA knock-in mice at disease onset, improving life expectancy and motor performances, which correlated with decreased mutant protein phosphorylation and accumulation (Polanco et al., 2016). In addition, the PACAP analog rescued SBMA-induced mitochondrial membrane depolarization in patient-derived neuroprogenitor cells, consolidating the therapeutic potential of PACAP and its derivatives. Moreover, electrophysiological phenotypes were investigated in SBMA MN-derived cells (Jimenez Garduno et al., 2017). Altered ionic currents were recorded in MN-derived cells expressing polyglutamine-expanded androgen receptor, and those abnormalities were partially rescued by PACAP treatment. These insights highlight a possible therapeutic application for PACAP in MNDs, such as ALS and SBMA.

Taken together, knowledge on the unique features of distinct types of neurons differentially vulnerable to MNDs is crucial for the development of novel therapeutic strategies and to deeply understand the mechanisms of neuroprotection of already developed drugs.

## Author Contributions

MP and EZ coordinated the work, wrote the manuscript, and provided the funding. DP and MB wrote and revised the manuscript. All authors contributed to the article and approved the submitted version.

## Conflict of Interest

The authors declare that the research was conducted in the absence of any commercial or financial relationships that could be construed as a potential conflict of interest.

## Publisher’s Note

All claims expressed in this article are solely those of the authors and do not necessarily represent those of their affiliated organizations, or those of the publisher, the editors and the reviewers. Any product that may be evaluated in this article, or claim that may be made by its manufacturer, is not guaranteed or endorsed by the publisher.
